# Analgesic efficacy of tramadol in cats with naturally occurring osteoarthritis

**DOI:** 10.1371/journal.pone.0175565

**Published:** 2017-04-12

**Authors:** Beatriz P. Monteiro, Mary P. Klinck, Maxim Moreau, Martin Guillot, Paulo V. M. Steagall, Jean-Pierre Pelletier, Johanne Martel-Pelletier, Dominique Gauvin, Jérôme R. E. del Castillo, Eric Troncy

**Affiliations:** 1 GREPAQ (Animal Pharmacology Research Group of Quebec), Faculty of Veterinary Medicine–Université de Montréal, Saint-Hyacinthe, QC, Canada; 2 Osteoarthritis Research Unit, Research Center of the University of Montreal Hospital Centre, Montreal, QC, Canada; 3 Department of Clinical Sciences, Faculty of Veterinary Medicine–Université de Montréal, Saint-Hyacinthe, QC, Canada; University of Bari, ITALY

## Abstract

**Objectives:**

This study aimed to (1) compare outcome assessments in normal and osteoarthritic cats and (2) evaluate the analgesic efficacy of tramadol in feline osteoarthritis (OA), in a prospective, randomised, blinded, placebo-controlled, crossover design.

**Methods:**

Twenty cats were included after clinical examination, blood work and full body radiographs were performed. In Phase 1, outcome assessments aimed to differentiate normal (*n* = 5; *i*.*e*. exempt of any radiographic and clinical sign of OA) from OA (*n* = 15) cats. In Phase 2, OA cats were treated twice daily with a placebo (PG: cornstarch 15 mg) or tramadol (TG: 3 mg/kg) orally for 19 days, with a 3-month washout period between treatments. Evaluations were performed in normal and OA cats at baseline and consisted of: 1) peak vertical force (PVF) after staircase exercise; 2) telemetered night-time motor activity (NMA); and 3) response to mechanical temporal summation (RMTS). After treatment, PVF, NMA and RMTS evaluations were repeated in OA cats. Data were analysed with mixed model methods with an alpha-threshold of 5%.

**Results:**

Phase 1: 1) PVF (% of body weight; mean ± SD) was higher in normal (59 ± 10.5) than in OA cats (50.6 ± 5.7) (*p* = 0.005); 2) NMA (no unit) was not different between groups; 3) RMTS (number of stimuli; median (range)) was higher in normal [29.5 (23.5–30)] than in OA cats [14 (8.5–28)] (*p* < 0.0001). Phase 2: PVF, NMA and RMTS presented a treatment effect (*p* = 0.024, *p* = 0.008 and *p* = 0.018, respectively). No clinically important adverse-effects were observed.

**Conclusion:**

Outcome assessments such as kinetics (PVF) and evaluation of central sensitisation (RMTS) are discriminant of OA status. Mobility measured by NMA was not discriminant of OA status, however it increased in OA cats with tramadol treatment. Nociceptive hypersensitivity quantified by RMTS was evident in OA cats and was responsive to tramadol treatment.

## Introduction

Osteoarthritis (OA) is a degenerative disease associated with pathological changes of the synovial joint. The progressive deterioration of one or more components of the joint is associated with pain, inflammation, peripheral and/or central sensitisation and decreased mobility, which ultimately impact activity and quality of life [1–5]. Radiographic evidence of OA is reported in up to 61% of cats older than six years of age [6] and in up to 90% of cats older than 12 years of age [7]. Similarly, the incidence of OA increases with age and is the leading cause of disability due to pain in both dogs [8] and humans [9]. In feline clinical practice, the signs of OA are very subtle and unspecific [10], and client-based questionnaires and activity monitoring have been used to assess pain-induced behaviours in osteoarthritic cats [6,11,12]. In the research setting, OA-related outcome assessments have been recently validated to characterise functional disability and maladaptive pain secondary to central sensitisation [3,4,13,14]. Indeed, brain functional imaging in cats with OA revealed sustained ascending nociceptive inputs and increased activity of the descending modulatory pathways, both consistent with central sensitisation [14]. The analgesic treatment of OA in cats has been classically based on the use of non-steroidal anti-inflammatory drugs (NSAIDs), such as meloxicam [2,3,11,15,16]. This compound seems to improve motor activity [3,11], but not central sensitisation [3,5].

Tramadol is an analgesic used worldwide for its effects on improved physical function and good tolerability in humans with chronic OA pain [17]. Nevertheless, evidence of its efficacy in canine and feline OA are scarce. The mechanisms of action of tramadol have not been fully elucidated and to date, the majority of studies have focused on the activation of μ-opioid receptors and inhibition of monoamine reuptake as potential mechanisms [18–20]. The analgesic effects of tramadol are expected to be mostly related to the production of its active metabolite(s) such as *O*-desmethyl tramadol (M1), which binds to μ-opioid receptors with approximately 300-fold higher affinity than the parent compound [19,21]. However, the affinity of tramadol for the μ-opioid receptor is very low, approximately 10-fold less than that of codeine and 6000-fold less than that of morphine. Yet, the increases in pain thresholds induced by tramadol differ from those of other opioids in that they are only partially blocked by naloxone [19]. These latter findings indicate that μ-opioid receptor activation is only one of the mechanisms of action of tramadol and M1. Other mechanisms of action include 1) inhibition of norepinephrine and serotonin (5-hydroxytryptamine (5-HT)) reuptake, allowing these central monoamine neuromediators to be more active in accentuating endogenous inhibitory control; 2) inhibition of G-protein coupled receptors such as α_2_-adrenoceptor, neurokinin 1 receptor, muscarinic receptor; and 3) inhibition of ion channels *via* nicotinic acetylcholine receptor and N-methyl-D-aspartate receptor [22]. These mechanisms of action can increase the activity of the endogenous inhibitory control and decrease the pain transmission likely explaining the central analgesic effects of tramadol.

In cats, the drug has high bioavailability after oral administration (93 ± 7%) and M1 follows tramadol’s disposition profile [23,24]. Indeed, studies indicate that cats might have superior analgesic profile after tramadol administration when compared with dogs due to a longer elimination half-life and higher concentrations of M1 [23,25]. Its effects in cats have been evaluated for the treatment of acute post-operative pain and in antinociceptive studies [20,24,26]. Tramadol is a low cost outpatient oral analgesic that is potentially a viable option for the treatment of OA pain, however, its efficacy for the treatment of feline maladaptive pain is not known.

The authors hypothesised that 1) assessments of peak vertical force (PVF) kinetics, night-time motor activity (NMA), as markers of functional disability, and response to mechanical temporal summation (RMTS), as marker of maladaptive pain secondary to central sensitisation, would differentiate healthy from OA cats, and 2) in OA cats, tramadol treatment would improve assessments of PVF, NMA and RMTS when compared with placebo treatment in a prospective, randomised, blinded, placebo-controlled, crossover design study. Results of this study suggest that cats with and without OA can be distinguished using objective outcome measures, and that cats with maladaptive pain seem to benefit from tramadol treatment, when compared with placebo treatment.

## Material and methods

### Animals and experimental protocol

The study was approved by the Institutional Animal Care and Use Committee (Comité d’Éthique et d’Utilisation des Animaux–n° Rech-1482) and animals were handled and housed according to the Canadian Council on Animal Care Guidelines. Furthermore, this study adhered to the the guidelines of the Committee for Research Ethical Issues of the IASP [27], and the ARRIVE guidelines for reporting animal research [28].

According to the inclusion criteria, experimental cats were selected based on normal physical and neurological examination and normal clinical pathology evaluations (complete blood count, serum total thyroxine (T4), serum chemistry profile and urinalysis). Whole body computed radiographs were performed under heavy sedation using medetomidine (0.02 mg/kg; Domitor 1 mg/mL, Zoetis Canada Inc., Kirkland, QC, Canada) and morphine (0.2 mg/kg; Morphine Sulfate Injection 10 mg/mL, Sandoz Canada Inc., Boucherville, QC, Canada), administered intramuscularly. Images were analysed by a board-certified radiologist [13]. Exclusion criteria included the administration of either an NSAID or a glucocorticoid within four or eight weeks, respectively, of the start of the study. An orthopaedic examination was performed in all animals. Two populations of cats were selected (*n* = 15 OA cats, and *n* = 5 normal cats). Geriatric cats (≥ 10 years of age) that presented radiographic signs of OA affecting at least one appendicular joint, and young adult cats (≤ 4 years of age) that did not present clinical or radiographic signs of OA, were assessed. For selection of OA cats, seventy-three cats from a colony of research animals normally used for investigations related to cognitive function were screened. None of the cats had experimentally induced orthopaedic disease. They also required being friendly and interested in human interaction, because they would be subject to constant handling during the study.

The cats were housed together in one large room with heat and humidity control, and with access to windows. Environmental enrichment was achieved with the use of toys, scratch posts, condos, bedding and covers. Cats were fed according to the food manufacturer’s recommendations twice daily with a standard certified commercial cat food (Hill’s Prescription Diet w/d Feline, CDMV, Inc., St.-Hyacinthe, QC, Canada). Fresh water was available *ad libitum* in fountains.

Prior to the beginning of the study, cats were acclimatised to the personnel, research facility and evaluation tools over 6 weeks. The study was divided into two phases. In Phase 1, OA (*n* = 15) and normal (*n* = 5) cats underwent three outcome assessments: PVF, NMA and RMTS. The evaluators were blinded to the OA-status of the cat. In Phase 2, after baseline measurement, OA cats were randomised to receive one of the following treatments twice daily for 19 days by the oral route: placebo (15 mg cornstarch) (PG: placebo group; *n* = 14) or tramadol (3 mg/kg; Tramadol HCl, Gentès & Bolduc Pharmaciens, Inc., St.-Hyacinthe, QC, Canada) (TG: tramadol group; *n* = 14). Treatments were repeated in a crossover design after a three-month washout period. Tramadol capsules were prepared individually based on the body weight (BW) of each cat in an attempt to be as close as possible to a dose of 3 mg/kg. Placebo and tramadol capsules were identical, and the evaluators were blinded to the treatments. Phase 2 evaluations included NMA and RMTS and were repeated at the end of the treatment period. Unfortunately, PVF evaluation was partially completed only during the Phase 2, for technical reasons (see below).

### Measurement of PVF

The PVF was acquired using a floor mat-based plantar force measurement system (Walkway System WE4, Tekscan) and was managed using Walkway Research Software v.7.0. Calibration was performed prior to each measurement. The cats were coaxed using positive reinforcement (treats, clicker, brushing, *etc*.) to trot across the walkway at a comfortable speed (0.8–1.4 m s^-1^) [29]. Speed was computed using the time and distance of a given trot. Only the four-foot strikes of the first stride of the trot were used for data comparisons. Among all kinetic gait parameters generated, only the maximal loading (*i*.*e*. PVF) was used [3,13,30].

For each evaluation session, the cat was allowed 10 trot attempts across the mat in order to provide three valid sets of four-foot strikes that would be used for data comparison. Furthermore, a valid attempt was defined as one in which the cat trotted across the entire mat undisturbed, consistently, in a straight line, and at a constant speed. If three valid trots had not been achieved after a maximum of 10 trot attempts, the cat was released and data for that cat were not used for comparison. The PVF was recorded over approximately 3 minutes immediately after 60 seconds of stairs exercise that consisted of running up and down a 10 meter staircase (S1 File).

Based on the PVF expressed as a BW percentage (% BW), the limb (thoracic or pelvic) that yielded the lowest PVF value in baseline assessment was determined for each cat. The outcome was calculated by averaging the three valid attempts of the most affected limb of each cat [3,30]. Unfortunately, the system was not operational for the second arm of the crossover, and could not be repaired. In consequence, PVF assessment during the Phase 2 could be completed only for about half of the OA cats.

### Motor activity assessment

The NMA was assessed using a collar-attached accelerometer-based activity sensor (ActiWatch, Minimitter/Respironics, Bio-Lynx Scientific Equipment, Ville-St.-Laurent, QC, Canada) maintained in place from 14 days prior to beginning of Phase 1, until the last day of evaluation of Phase 2 (S2 File). The device was set to perform one activity intensity count every 2 minutes. The amplitude of each count was subsequently translated to a numeric value (from 0 to infinite) referring to the intensity count of MA. Based on previous research [3,13], in order to avoid any interference caused by human interaction, only data from Friday, Saturday and Sunday evenings (from 17:00 to 06:58 hours) were used for NMA analyses. The collars were checked twice daily to ensure that the activity sensors were in place. For each period, the mean of the intensity counts was calculated for each cat and used for data comparison.

### Response to mechanical temporal summation

Repeated mechanical stimuli of sub-threshold intensity at a fixed intensity (4 N), frequency (0.4 Hz) and duration (up to 30 stimulations over 75 seconds) were applied using a purpose-made device (Topcat Metrology Ltd; Cambs, UK). The mechanical stimulus was produced by a metallic pin (10 mm long) with a hemispherical tip (2.5 mm in diameter) mounted on a rolling diaphragm actuator that was placed on the cranial aspect of the right or left mid metacarpus, held by a narrow band around the limb. A ‘dummy’ device was placed on the contralateral limb. This pin would move back and forth perpendicularly to the skin, producing a pressure stimulus at each time (similarly to a repetitive mechanical nociceptive stimulation). The number of stimulations needed to elicit a behavioural response was recorded (S1 File). Increases in the number of stimulations are considered to reflect decreases in central pain hypersensitivity. The evaluation protocol is described elsewhere [4,5].

### Adverse effects

Cats were monitored daily for any potential outwardly detectable treatment-induced adverse effects. Attitude (normal, depressed/sedated or euphoric) and pupil size (normal or dilated) were evaluated during treatment administration (every 12 hours). In addition, at any time that a clinical sign was witnessed by a staff member, the date, time, affected cat and clinical sign were recorded. Therefore, each time that a cat was noted to present with an abnormality, it was considered as ‘one event’ (*i*.*e*. if a cat was evaluated as ‘euphoric’ twice in one day, then two events of ‘euphoria’ were added to the total number of events). Clinical pathology evaluations, including complete blood count, serum chemistry profile, total T4 and urinalysis, were performed at baseline and repeated after the last treatment (day 19).

### Statistical analyses

According to hypotheses, analyses on PVF and NMA were two-sided, and those on RMTS were one-sided, using an α-threshold value of 0.05. Analyses were carried out using sas version 9.3 (SAS Institute, Inc., Cary, NC, USA). The distribution of continuous data was assessed using the Shapiro-Wilk test (normal distribution) or kernel density estimation. The PVF and NMA data were assumed to be log-normally distributed, and the RMTS count data were assumed to be Poisson-distributed by nature. The Phase 1 comparisons between OA and normal cats were made using a generalised linear model for PVF and RMTS assessments, and an exact Wilcoxon-Mann-Whitney test for NMA assessments. The Phase 2 treatment effect was evaluated using a generalised linear mixed model for repeated measures with treatment groups, time and their interaction as fixed effects, and cat as a random effect. For each model, the homogeneity of variances was tested, and the best structure of the covariance was assessed using information criteria that measured the relative fit of the competing covariance model. Then, the treatment and time effects were assessed using likelihood modeling. More precisely, for PVF, it used a compound symmetry covariance structure test with BW, velocity and maximum number of attempts as covariates, NMA a paired *t*-test, and RMTS a signed rank test.

## Results

Seven female and eight male cats were included in the OA group, and two female and three male cats were included in the normal group. All animals were neutered and completed the investigation, except for two OA cats that were withdrawn due to reasons unrelated to the study. During the wash-out period, one cat developed skin allergies that required further investigation, and another cat had to be euthanised due to stage III chronic kidney disease. In the first period of the crossover, these latter cats had been included in the TG (*n* = 8) and PG (*n* = 7), respectively. Thus, in the second period of the crossover, there were *n* = 6 cats in TG and *n* = 7 cats in PG. These exclusions resulted in available data for *n* = 14 cats in each TG and PG at the end of the study. In the OA group, cats had radiographic signs of OA in the unilateral hip (*n* = 4), bilateral hips (*n* = 2), shoulders (*n* = 3), elbows (*n* = 2), combination of tarsus and elbow (*n* = 1), or combination of shoulders and hips (*n* = 3). At inclusion, the ranges of age and BW of OA cats were [10.17–11.75] years, and [2.91–6.05] kg, respectively. In the normal cats’ group, respective ranges were [2.71–3.92] years, and [3.08–5.23] kg.

Findings of Phase 1 are presented in Table 1. PVF data were not available for three OA cats due to reluctance to complete the assessment. PVF was higher in normal cats when compared with OA cats (*p* = 0.005). Data for NMA were not available for one normal cat due to a defective device (battery failure). Night-time MA was not different between groups. The RMTS was higher in normal cats when compared with OA cats (*p* < 0.0001).

**Table 1:** Peak vertical force (PVF), night-time motor activity (NMA) and response to mechanical temporal summation (RMTS) in cats with and without naturally-occurring osteoarthritis (OA and normal cats, respectively).

	OA cats		Normal cats	
	*n*	Mean (SD) or Median [Min–Max]	*n*	Mean (SD) or Median [Min–Max]
**PVF (% BW)**	12	50.6 (5.7)	5	59.0 (10.5)*
**NMA (no unit)**	15	47.8 (21.4)	4	58.3 (38)
**RMTS (number of stimulations)**	15	14 [8.5–28.0]	5	29.5 [23.5–30.0]*

* Significant between-group difference.

**Fig 1:**
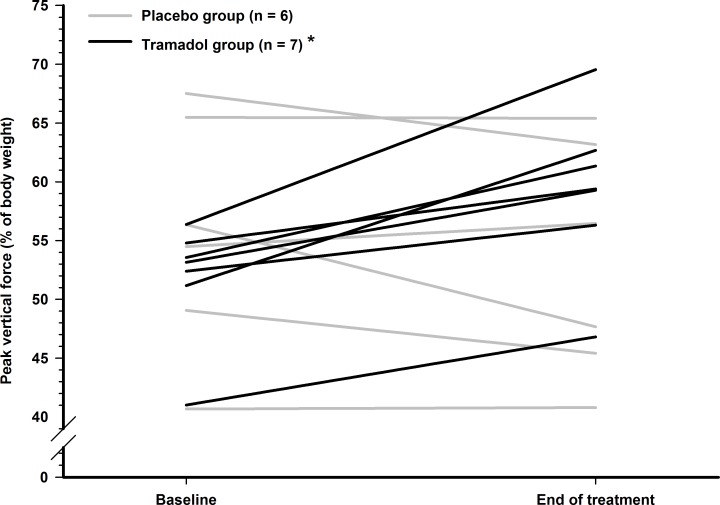
Individual values of peak vertical force before and after treatment in osteoarthritic cats. A pressure-sensitive mat was used for collection of data. Each value is the average of three valid attempts of the most affected limb of each cat. Cats with OA were randomly divided into two groups to receive either placebo (*n* = 6) or tramadol (*n* = 7; 3 mg/kg every 12 hours orally) and were re-evaluated after 19 days of treatment. *Significant between- and within-group difference.

After 19 days of treatment, PVF was collected for seven cats in TG, and six cats in PG (Fig 1), as a result of reluctance to complete the assessment for two cats, and technical failure of the system during the second arm of the crossover.

**Fig 2:**
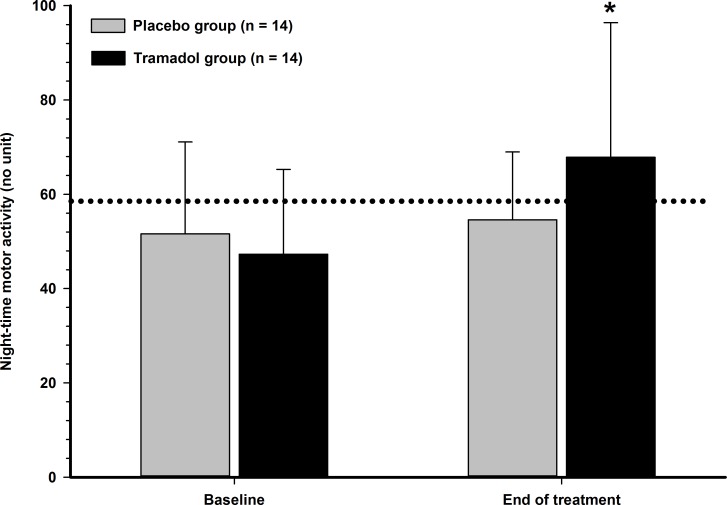
Night-time motor activity in cats with naturally occurring osteoarthritis. A collar-attached accelerometer device was used for collection of data. Cats with OA (*n* = 14) were randomly divided into two groups in a crossover design to receive either placebo or tramadol (3 mg/kg) every 12 hours orally and were re-evaluated after 19 days of treatment. Values are presented as mean (SD). The dotted line represents the averaged night-time motor activity (no unit) observed in normal cats during baseline evaluations. *Significant between- and within-group difference.

From baseline to end of treatment, PVF increased in all TG cats (*p* = 0.012), and this increase was considered important (> 10% of change percentage) in 5/7 cats, whereas none (0/6) of the PG treated cats showed improvement (*p* = 0.235). The range of percentage of change for the TG and PG were [+7.5%–+23.4%] and [-15.4%–+3.6%], respectively. The treatment effect was significant (*p* = 0.024), even with such a limited sample size. The NMA was significantly different between treatment groups at the end of treatment (*p* = 0.008) and activity only increased in the TG cats (Fig 2).

**Fig 3:**
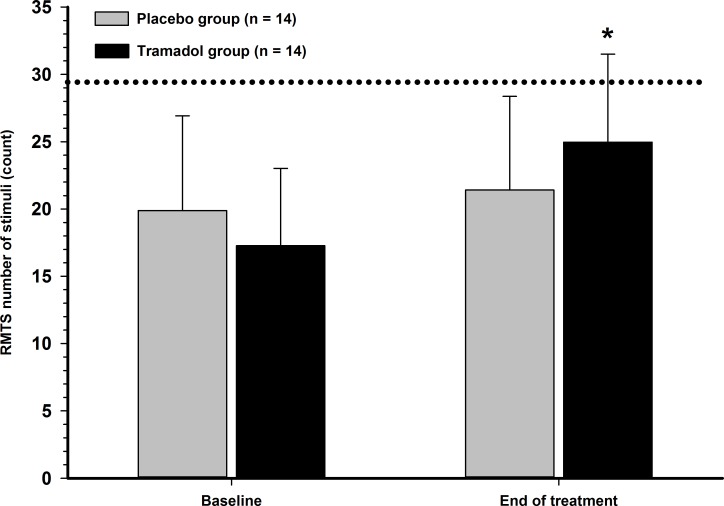
Response to mechanical temporal summation (RMTS) in cats with naturally occurring osteoarthritis. A mechanical device was used for collection of data. Cats with OA (*n* = 14) were randomly divided into two groups in a crossover design to receive either placebo or tramadol (3 mg/kg) every 12 hours orally and were re-evaluated after 19 days of treatment. Values are presented as mean (SD). The dotted line represents the number of mechanical stimuli (count) observed in normal cats during baseline evaluations. A significant between-group difference (MWW test) was found between normal cats and cats with osteoarthritis. *Significant between- and within-group difference.

The RMTS was also significantly different between treatment groups at the end of treatment (*p* = 0.018) (Fig 3). The cut-off value (30 stimulations) was not reached in any OA cat at baseline. Six TG and three PG cats achieved the cut-off at the end of treatment.

No adverse systemic effects were observed with either treatment based on clinical pathology evaluation. The following outwardly detectable adverse effects were recorded (treatment group: number of events): mydriasis (TG: 32; 20 events were related to *n* = 3 cats and PG: 3), mild sedation (described by observers as “depressed” or “overly quiet”) (TG: 32; 29 events were related to *n* = 2 cats), mild euphoria (TG: 6), polydipsia (TG: 1 and PG: 1), vomiting (TG: 8; 7 events related to *n* = 2 cats and PG: 1).

Although not systematically assessed, most cats allowed a stress-free pill administration with the use of a pet pillar device, with the exception of 3 cats, which displayed aversive and hiding behaviour (unknown frequency), hypersalivation (TG: 9 and PG: 2) and blood in the mouth (TG: 6 and PG: 1, all events related to the same cat) during pilling.

## Discussion

Objective outcome measures such as PVF and RMTS were discriminant of OA status and revealed decreased biomechanical function and increased nociceptive hypersensitivity in OA cats, respectively. Night MA does not seem to differentiate between cats with and without OA, and perhaps a larger sample size would overcome the inter-individual heterogeneity observed. Treatment with tramadol demonstrated beneficial effects by increases in PVF, NMA and RMTS when compared to placebo treatment. There were no clinically important adverse-effects.

Assessment of PVF characterises biomechanical activity and is a valid and reliable tool for evaluating chronic pain in cats with OA [3–5,13,29,30]. Similar to previous studies evaluating cats with hip OA [13], PVF values in Phase 1 were higher in normal cats when compared with OA cats. This may reflect altered biomechanics in OA pathology and/or decreased weight-bearing in affected limb secondary to pain. Nevertheless, it may also reflect neuromuscular aging-related changes to the musculoskeletal system such as decline in passive joint stability, ligament stiffness and muscle strength [31]. In fact, PVF values have been negatively correlated with age in feline patients [13]. It would have been ideal to have PFV data of all OA cats during Phase 2 in order to clarify whether the differences observed in Phase 1 are related to age only or OA status. Unfortunately, this was not possible due to technical limitations and we only have PVF data for a few cats in the first treatment period. It has to be noted that the most affected limb PVF, as used, could refer to either thoracic or pelvic limb, increasing the intra-group variability in collected data. Indeed, in several quadrupeds, the normal weight distribution during trot is higher in the thoracic (55% for cats) than in the pelvic (45% for cats) limbs [29]. The center of mass being closer to the thorax contributes to this imbalance [32]. Consequently, the range of values for the thoracic PVF is higher than for the pelvic PVF, and the intra-group variability of the most affected limb PVF, when both thoracic and pelvic limbs are considered, is higher than the one restricted to PVF of thoracic or pelvic limbs alone, in OA-affected cats. Therefore, it is understood that the unfortunately limited sample size in Phase 2, evaluating treatment effect, had greatly reduced the power of inferential analysis. It is difficult to ascertain with great confidence that changes in PVF assessment are related to a treatment effect and not a type-I statistical error. Furthermore, placebo-treated cats may show exercise-induced improvement in PVF [3,5] due to all the necessary acclimation and training involved with PVF assessment in the species [13,30]. In these previous studies and the study herein, cats required a minimum of four weeks of training which inherently increased their physical activity. Thus, improvements in PVF assessment in placebo-treated cats might reflect the benefits of exercise alone in the management of OA.

Accelerometer-based MA provides an objective assessment of functional disability related to OA-associated maladaptive pain. This method has been largely used as an outcome to evaluate mobility and the impact of chronic pain in the quality of life of cats in the research and clinical settings [3–5,11–13,33]. Accelerometer-based MA had very good intra-class correlation coefficient indicating a good reliability of the test [3]. We had hypothesised that NMA of normal cats would be higher when compared with OA cats in Phase 1, as it has been previously reported [13], however we could not find such a difference. One must take into consideration that this assessment is closely related to the individual’s natural behaviour [3–5,11–13,33] and it may be that cats in the normal group were naturally less active. In addition, the number of cats in each group was unequal (5 *versus* 15 cats) and a large intra-group variability might have existed which would reduce the power to find a statistical difference. We speculate that if the number of individuals were similar, a difference would have been detected and would have corrected a type-II statistical error. Nevertheless, assessment of NMA revealed a clear treatment-effect, which is in agreement with other reports with tramadol [5], or the NSAID meloxicam [3,5,11,12], or even a veterinary OA therapeutic diet [33]. The mechanism by which this phenomenon takes place is unclear, but most likely it reflects an analgesic effect of tramadol and consequent increased mobility. In humans, tramadol is recognised for increasing mobility [17]. Considering that exercise is one of the most important pillars in OA-treatment and directly reflects quality of life, it seems reasonable to say that by increasing MA, tramadol could play an important role in improving the quality of life of osteoarthritic cats.

Central sensitisation is expressed as pain hypersensitivity characterised by decreased tactile (von Frey) threshold [3,4], decreased RMTS [4], sustained cerebral nociceptive inputs (secondary somatosensory cortex) and increased activity of descending modulatory pathways (thalamus and periaqueductal gray matter) [14] in cats with OA. Temporal summation is considered to be an important tool for the study of maladaptive pain as it reflects the early phase of central sensitisation (“wind-up”). The latter is an intrinsic part of the early neuroplastic changes in the central nervous system [34]. This phenomenon is potentially reversible and tramadol has been used for this purpose in animals and humans [35,36]. The RMTS was lower in OA when compared with normal cats in Phase 1, which was similar to another study [4]. In OA cats, RMTS increased from 14 stimulations at baseline evaluation (median value in Table 1) to 25 stimulations after tramadol treatment and a difference between TG and PG was found (Fig 3). These findings would infer that OA and normal cats present different neuro-sensitivity profiles and that OA cats are affected by central sensitisation, which in turn is translated by low RMTS. Thus, tramadol might reduce central sensitisation, alone (this study) or in combination with meloxicam [5], in OA-affected animals by means of RMTS, which was not observed in placebo-treated cats.

No clinically important adverse-effects were recorded. Mydriasis, sedation, euphoria and vomiting may be expected in cats after tramadol treatment [36]. Sedation was recorded mostly in the same *n* = 2 cats in TG, but did not seem to be important enough to affect activity as measured by NMA assessment. Indeed, as observers described sedation as cats being “overly quiet”, the latter may be related to the personality of those 2 individual cats. Hypersalivation and blood in the mouth were more frequent in TG and are likely related to reluctance to pill administration. Tramadol is bitter tasting and may cause hypersalivation and retching if the capsule breaks open and the animal tastes the drug [37]. The bitter tasting of tramadol may become an obstacle to the treatment of some individuals as observed herein. Furthermore, the presence of blood in the mouth might also be related to the presence of stomatis-gingivitis complex in these cats that would make them more likely to develop gingival bleeding after administration of the medication.

Antinociceptive studies suggest that a dosing regimen of 4 mg/kg administered orally four times daily would be ideal [24]. In the study herein, because long-term treatment would be administered and no data was available for clinical cats, a dose of 3 mg/kg was chosen. The drug was administered every 12 hours to simulate intervals of administration that are commonly prescribed to owners for convenience and increased compliance. Moreover, it was the same dose used in a previous study [5], where tramadol was combined to meloxicam oral transmucosal spray.

## Conclusion

Based on PVF and RMTS, normal and OA cats display different biomechanics and central sensitisation profile. Assessment of NMA was not discriminatory of OA-status. Treatment with tramadol increased weight-bearing, mobility and decreased central sensitisation based on PVF, NMA and RMTS in cats with naturally occurring OA. To the best of our knowledge, this is the first demonstration of clear benefits of tramadol treatment in cats with OA. Long-term tramadol therapy of up to 19 days seems safe and most common adverse-events are mydriasis, sedation and euphoria. These results are encouraging for promoting tramadol as a treatment for pain in osteoarthritic cats.

## Supporting information

S1 File Raw data for Peak Vertical Force and Response to Mechanical Temporal Summation outcomes. The excel file includes all raw data of Peak Vertical Force and Response to Mechanical Temporal Summation outcomes measurement for normal (*n* = 5) and osteoarthritic (*n* = 15) cats used in Phase 1, as well as all raw data for the 15 osteoarthritic cats used in Phase 2.
(XLSX)

S2 File Raw data for Motor Activity outcome. The excel file includes all raw data of Motor Activity outcome measured (epoch of 2 min) for normal (*n* = 4) and osteoarthritic (*n* = 15) cats used in Phase 1 and Phase 2.
(XLSX)
